# A Cost-Effective and Robust Cell-Based Bioassay Method for Evaluating the Bioactivity of Trastuzumab-like Antibodies

**DOI:** 10.3390/biomedicines13010023

**Published:** 2024-12-26

**Authors:** Pooja Bharali, Subhash Chand, Harish Chander

**Affiliations:** National Institute of Biologicals, Ministry of Health & Family Welfare, Govt. of India, A-32, Sector-62, Noida 201309, UP, India; poojabharali02@gmail.com

**Keywords:** trastuzumab, biosimilars, anti-proliferation assay, biological activity

## Abstract

**Background/Objectives:** Trastuzumab is an effective therapeutic intervention for treating HER2-positive breast cancers. The cost-effectiveness, global demand, and patent expiration of trastuzumab have led to the inflow of its biosimilars in the global market. With the rise of biosimilars in the biopharmaceutical market, it has become crucial to ensure that the biosimilar is at par with the original monoclonal antibody (mAb)in terms of efficacy, safety, and quality. Bioassay is one of the critical quality attributes (CQAs), hence developing a reliable and robust bioassay is essential for the evaluation of their biological activity and the harmonization of the quality of these biologics, supporting their safe and effective use in clinical practice. **Methods:** The present study aimed to develop a robust cell-based bioassay to assess the bioactivity of trastuzumab and its biosimilars for quality control testing. For this purpose, molecular characterization of different HER2-positive breast cancer cell lines of SKBR3, BT474, MDA-MD-453, MDA-MB-175, MCF-7, and MDA-MB-231 was performed to select a suitable cell line for the cell-based bioassay. **Results:** The SKBR3 cell line was found to express the HER2 receptors significantly higher in comparison to the other cell lines, and it was thereby selected for further bioassay optimization. The biological activity of trastuzumab was determined using the inhibition of proliferation (IOP) assay on the SKBR3, which was optimized based on the parameters of cell seeding density, drug dilution range, and incubation time, and it was further validated as per the compendial guidelines and found valid for the parameters of specificity, accuracy (% relative bias = 0.0067%), precision (repeatability: % GCV = 1.21%), linearity (R2 = 0.99), and range (50% to 200%). Additionally, the biological activity of different trastuzumab biosimilars was assessed using the validated IOP assay and compared to the HER2 binding assay performed by flow cytometry. The biological activity of different trastuzumab biosimilars was found to be comparable to the WHO primary reference standard of trastuzumab in terms of its relative potency using the IOP assay and binding assay by flow cytometry. **Conclusions:** Thus, an economic and robust cell-based bioassay method was successfully developed to assess the bioactivity of trastuzumab and its biosimilars.

## 1. Introduction

Monoclonal antibodies represent a revolutionary class of biotherapeutic molecules that have significantly impacted the treatment landscape of cancer and immunological disorders. Trastuzumab is a biotherapeutic monoclonal antibody that thwarts the signaling of human epidermal growth factor receptor 2 (HER2), and it is specifically employed in the treatment of breast and gastric cancers, characterized by high levels of HER2 expression [1]. Its administration encompasses both monotherapy and combination with various chemotherapies. It substantially enhanced the response rate and survival of patients with early-stage and metastatic breast cancers [2]. With increases in the incidences of breast and gastric cancers globally, there is an escalation in the demand for the biological drug. Biosimilar drugs are biotherapeutic products that closely resemble the original innovator biological molecule in terms of structure and biological activity, with no clinically significant differences in safety, purity, potency, and immune response [3,4]. Following the 2014 patent expiration of trastuzumab, Trastuzumab-dkst, developed by Mylan, USA (Ogivri) and Biocon, India (CANMab), became the first trastuzumab biosimilar to enter the global market, paving the way for subsequent trastuzumab biosimilars approved by regulatory agencies globally [5,6].

All biosimilars approved by the FDA, EMA, or WHO undergo a rigorous evaluation to ensure that they are on par with the innovator drug in terms of safety, potency, and quality [4]. The regulatory agencies EMA, ICH, and WHO lay down regulatory guidelines which ensure that the biosimilar drug products are effective and safe based on a comprehensive, head-to-head biosimilarity assessment study that includes analytical testing to establish structural and functional similarity [7]. The product’s attributes crucial for the safety, effectiveness, and strength of the biosimilar drug product are categorized as critical quality attributes (CQAs) [3,7]. These assessments are conducted to establish comparability between the reference product and its intended biosimilar, a process known as similarity assessment (or biosimilarity). This process entails an in-depth comparative analysis of structural and functional characteristics utilizing validated analytical methods [8]. Evaluation of biological activity is an important CQA for the functional characterization of biosimilar drugs. The bioactivity of an antibody drug is equivalent to the potency of a biological drug. Biological activity is a critical quality attribute for biosimilar development and was required to be performed routinely at a national control laboratory and by the manufacturer before batch release [9,10]. A reliable and robust cell-based assay is essential to determining the in vitro bioactivity of trastuzumab and its biosimilars at the developmental stage and during routine batch testing and post-market surveillance testing [10]. Further, a cell-based assay for the estimation of the biological activity of trastuzumab-like antibodies has an advantage over binding assays by ELISA or flow cytometry as it mimics the clinical mode of action by the phenotypic responses of cell proliferation or cell death [9,10,11]. Additionally, conventional cell proliferation or cell death assays are economical in comparison to flow cytometry-based assays or reported gene assays, considering the cost required to run a sophisticated machine like the flow cytometer and the cost of reported gene assay kits for routine batch testing [12]. Furthermore, developing a good potency assay is an arduous task due to challenges such as the innate variability and complexity in a biological system, diverse mechanisms of action, biological complexity of a drug product, as well as regulatory requirements. An ideal potency assay should be able to indicate the therapeutic potential of the antibody–drug in an in vitro setting as well as perform consistently in quality control environments. Moreover, a cell-based potency assay needs to be extensively validated and optimized as per international guidelines to ensure its reliability and accuracy in terms of critical assay parameters of specificity, linearity, detection range, precision, and accuracy [13,14].

There has been a colossal influx of biosimilar drugs in the global market after the expiration of originator products, which has made them the subject of stringent regulatory scrutiny. As a result, economical and robust cell-based assays are desired not only by product manufacturers but also by national control laboratories to ensure the harmonized quality of their biosimilars throughout the product’s life cycle. The present investigation aimed to develop an economical and robust cell-based bioassay platform using the molecular expression of HER2 receptors to assess the biological activity of biosimilars of anti-HER2 trastuzumab-like therapeutic monoclonal antibodies in in vitro settings. To achieve the objective, the initial phase of this study involved the screening of a HER2-positive cell line characterized by the maximum overexpression of the HER2 receptor on its surface. Subsequently, efforts were directed toward optimizing and validating the anti-proliferation assay of trastuzumab biosimilar drugs on the selected cell line. Furthermore, this study aimed to assess the biological activity of the trastuzumab biosimilars (N = 4) with the trastuzumab reference standard and comparison with a binding assay using flow cytometry, to determine the variability in biological activities using both the methods (IOP and binding assay by flow cytometry) and the suitability of the biosimilar’s adoption for routine batch testing by manufacturers and national control laboratories.

## 2. Materials and Methods

### 2.1. Materials

Four indigenously manufactured trastuzumab biosimilars approved in India were included in this study and the 1st WHO International Standard (NIBSC-19/108) [15] for the biological activity of trastuzumab, was used as the reference standard were listed in Table 1. To maintain confidentiality, the biosimilars were randomly coded and are not presented in the experiment in the order listed in Table 1.

### 2.2. Cell Lines and Culture Conditions

HER2-overexpressing breast cancer cell lines including SKBR3 (HER2-enriched, ATCC no-HTB-30), BT474 (Lum-B, ATCC no-HTB-20), MDA-MD-453 (HER2-enriched, ATCC no-HTB-131), MDA-MB-175 (HER2-enriched, ATCC no-HTB-25), MCF-7 (Lum-A, ATCC no-HTB-22), and MDA-MB-231 (TNBC, ATCC no-HTB-26) were procured from the American Type Culture Collection (ATCC, Rockville, MD, USA). The cells were cultured in DMEM (Gibco, Thermo Fisher Scientific, Inc., Carlsbad, CA, USA) with 10% FBS (Gibco, Thermo Fisher Scientific, Inc., Carlsbad, CA, USA), 100 µg/mL streptomycin, and 100 U/mL penicillin (Himedia laboratories, Maharashtra, India), and they were maintained at 37 °C in an incubator with 5% CO_2_ under humidified conditions.

### 2.3. RNA Extraction and RT-qPCR

Total cellular RNA was extracted from 1 × 10^6^ cells using the Qiagen QuickLyse Miniprep Kit (Qiagen N.V.). The cDNA was then prepared via reverse transcription of the purified RNA using the Bio-Rad iScript cDNA Synthesis Kit (Cat. No. 1708890, Bio-Rad Laboratories, Hercules, CA, USA). The thermocycler conditions were the following: 25 °C for 5 min, 46 °C for 20 min, and 95 °C for 1 min. Conventional quantitative PCR was carried out using TB Green Premix Ex Taq II (Cat. No. RR82LR, Takara, Shiga, Japan). Reactions were prepared in triplicate in 25 μL volumes using forward primers for HER2 (5′-TGTGGCTCTCAGATAATCAGTCC-3′) and GAPDH (5′-CTGGGCTACACTGAGCACC-3′) and reverse primers for HER2 (5′-AGCACCATTTTCTGGGTCTCT-3′) and GAPDH (5′-AAGTGGTCGTTGAGGGCAATG-3′) on a PCR System (CFX96, Bio-Rad Laboratories, Hercules, CA, USA). The thermocycler conditions were the following: initial denaturation of 95 °C for 5 min, and then 40 cycles of 95 °C for 10 s, followed by 58 °C for 30 s, and 72 °C for 30 s, with a final extension of 65 °C for 5 s. The data analysis involved utilizing the ΔΔCq method, with normalization against GAPDH [16]. MDA-MB-231 cell lines were taken as a negative control.

### 2.4. Immunoblotting

Cells were initially seeded in T-175 flasks, and then 1.0 × 10^7^ cells were trypsinized and harvested using ice cold 1× PBS (Invitrogen, Thermo Fisher Scientific, Inc., Carlsbad, CA, USA). To isolate the proteins, 250 µL of cell extraction buffer (Cat. No. FNN0011, Thermo Fisher Scientific, Inc., Carlsbad, CA, USA) was used along with 10 µL of protease inhibitor cocktail (Cat. No. P8340-1ML, Sigma Aldrich, St Louis, CO, USA). The BCA (Cat. No. 712853CN, EMD Millipore, Merck, San Jose, CA, USA) method was used to quantify the protein concentrations. The cells were then incubated for 30 min and later centrifuged at 14,000 × *g* for 20 min at 4 °C. A total of 40 µg of protein lysate samples were subjected to electrophoresis on 4% and 10% SDS-PAGE gel and then transferred to a nitrocellulose membrane. The nitrocellulose membrane was blocked for 2 h at room temperature with 5% BSA (Cat. No. 83803, SRL, India) in 1 × TBST. The membrane was incubated overnight at 4 °C with anti-HER2 antibody (1:1000, Cat. No. AHO1101, Thermo Fisher Scientific, Inc., Carlsbad, CA, USA) prepared in 1 × TBST and 3% milk, and anti-GAPDH (ZG003, 1:2000, Thermo Fisher Scientific, Inc., Carlsbad, CA, USA) prepared in 5% milk and 1 × TBST. A goat anti-mouse HRP-conjugated secondary antibody (1:1000, Cat. No. A21010, Abbkine, Santa Ana, CA, USA) was used for the membrane and incubated for 2 h at room temperature. Finally, the image was developed by ECL (Cat. No. 1715060, Bio-Rad Laboratories, Hercules, CA, USA) in the Chemidoc XRS+ system (Bio-Rad Laboratories, Hercules, CA, USA).

### 2.5. Inhibition of Proliferation (IOP) Assay

A total of 1.0 × 10^4^ SKBR3 cells/well were seeded in a 96-well plate and incubated for 24 h. Trastuzumab biosimilar drugs acquired from 4 different manufacturers and the reference standard (NIBSC-19/108) were added in two-fold dilutions in triplicate and in 3 plate settings at a concentration of 2 µg/mL. The plates were incubated for 5 days/120 h and 30 µL of alamarBlue (Cat. No. 199303, Sigma Aldrich, St Louis, CO, USA) was added to the cells and incubated for 8 h. Fluorescence was measured in a multimode plate reader (Spark, TECAN, Männedorf, Switzerland) at 530 and 590 nm wavelengths. A parallel line assay (PLA) was used to calculate the relative potency of the bioassay using PLA 2.1 software (Bioassay de Software, Rodgau, Germany) for statistical analysis.

### 2.6. Flow Cytometry

A total of 1.0 × 10^5^ cells were centrifuged then resuspended in 50 μL cold 1 × Dulbecco’s PBS (DPBS) buffer. The cells were rinsed and resuspended in the same buffer. Trastuzumab was added to the cells from a dose concentration of 20 to 0.04 μg/mL for 30 min at 4 °C. Samples were centrifuged at 290× *g* for 5 min at 4 °C, and pellets were resuspended in 50 μL of the 1 × DPBS containing anti-human IgG labeled with FITC-A (1:60, Cat. No. F9512, Sigma Aldrich, St Louis, CO, USA) and incubated for 30 min at 4 °C, with subsequent washing and centrifuging at 290 × *g* for 5 min at 4 °C. The pellets were then resuspended in 200 μL of 1 × DPBS and acquired in the flow cytometer (BD FACSLyric, BD Biosciences, Billerica, MA, USA). For the surface expression study for the HER2 receptor in different breast cancer cell lines, 50 μg/mL of trastuzumab was added to the cell lines SKBR3, BT-474, MDA-MB-453, MDA-MB-175, MCF-7, and MDA-MB-231 and acquired in the flow cytometer (BD FACSLyric, BD Biosciences, Billerica, MA, USA).

### 2.7. Validation of IOP Assay

The IOP assay validation was conducted according to the international pharmaceutical guidelines of the ICH (Validation of analytical procedures: text and methodology Q2 (R1)) [13], US Pharmacopeia (USP for development, validation, and analysis of bioassays, Chapters 1032, 1033, and 1034) [17,18,19], and European Pharmacopeia (Chapter 5.3 of Statistical Analysis of Results of Biological Assays and Test, European Pharmacopeia) [20] to assess the biological potency, which was a CQA of the biological drug. The key parameters evaluated for the assay were specificity, linearity, accuracy, and precision, encompassing repeatability and intermediate precision.

### 2.8. Statistical Analysis

All statistical analyses were conducted using GraphPad Prism 9 software (GraphPad Software, San Diego, La Jolla, CA, USA). For the bioassay, experiments were performed in triplicate and the slopes were plotted using the non-linear four-parameter logistic (4-PL) model in GraphPad Prism 9 software. For the relative potency analysis, PLA 2.1 software (Bioassay de Software, Rodgau, Germany) was used. An analysis of variance (ANOVA) with the F-distribution was used to test for the equality of slopes. GraphPad Prism 9 was used to analyze the potency data using one-way ANOVA followed by Dunnett’s test. Additionally, a two-tailed paired *t*-test was employed to analyze the two groups. A significance level of *p* < 0.05 was taken into consideration to designate a statistically significant difference.

## 3. Results

### 3.1. Selection of Cell Line for IOP Assay for Assessing the Biological Activity of Trastuzumab Biosimilar Drugs

**Figure 1:**
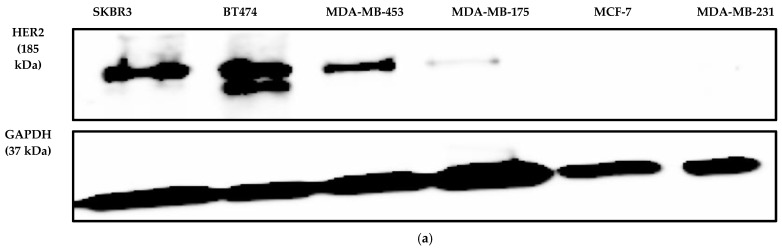

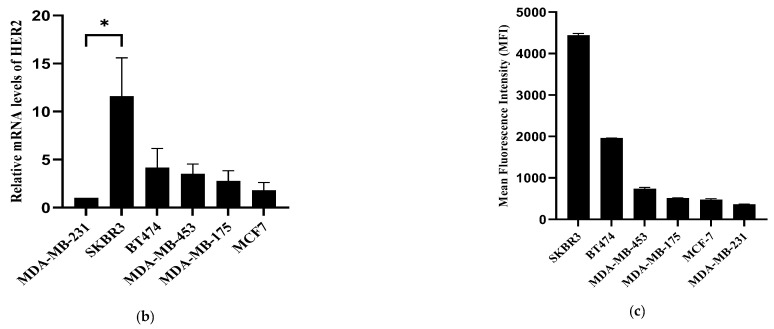
Screening of HER2-positive cell line for bioassay. (**a**) Immunoblotting analysis of HER2 receptor protein isolated from 6 cell lines. (**b**) Bar diagram of relative mRNA level expression of HER2 of 6 different cell lines. MDA-MB-231 was taken as a negative control for the experiment. Each data point is the average of three replicates and the error bars represent standard error. For the statistical analysis, the levels of HER2 receptor expressions in the HER2-positive cell lines were compared to the HER2-negative cell line of MDA-MB-231. There was a statistically significant difference at * *p* < 0.05. (**c**) Mean fluorescence intensity (MFI) measured by the flow cytometry of different cell lines when treated with the reference standard (NIBSC 19/108) to check the surface expression of HER2. Data are represented as the mean ± SD.

Various cell lines expressing the HER2 receptors were screened to assess the bioactivity of the trastuzumab biosimilar. Immunoblotting was performed to check the cell surface-level expression of HER2 receptor protein in the cell lines of SKBR3, BT474, MDA-MB-231, MDA-MB-175, MDA-MB-453, and MCF-7. Amongst the cell lines, it was found that SKBR3, BT474, and MDA-MB-453 expressed the HER2 receptor abundantly (Figure 1a). The relative mRNA levels of the HER2 gene were also measured in the cell lines, amongst which the SKBR3 cell line exhibited the highest expression of the HER2 gene followed by other cell lines (Figure 1b). Furthermore, an antigen-binding assay was performed to determine the receptor occupancy of the trastuzumab reference antibody drug as well as the surface expression of the HER2 receptor in different cell lines via flow cytometry. The results from flow cytometry demonstrated that the SKBR3 cells efficiently bound to the trastuzumab reference standard (NIBSC-19/108), exhibiting an MFI of 4224.5 as compared to other HER2-positive breast cancer cell lines BT474 with an MFI of 1961.5, MDA-MB-453 with an MFI of 740.5, and MDA-MB-175 with an MFI of 514 (Figure 1c). Therefore, SKBR3 cells were selected to further to optimize and validate the inhibition of the proliferation assay as well as to examine the biological activity of trastuzumab biosimilar drugs.

### 3.2. Optimization of IOP Assay for Assessing the Biological Activity on SKBR3 Cells

#### 3.2.1. Optimization of the Working Drug Concentration Range

**Figure 2:**
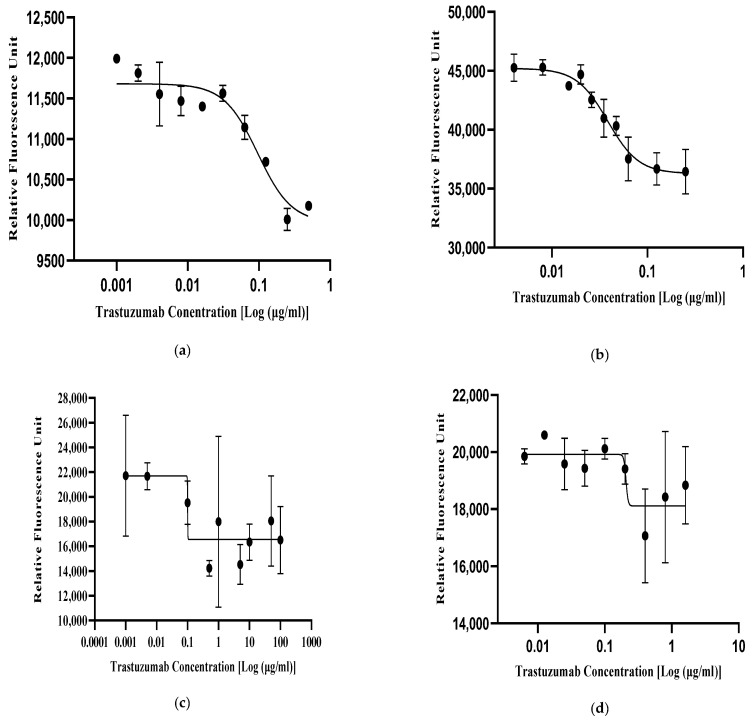
Optimization of the working dose concentration range. The 4-PL curve fitting for the trastuzumab reference standard (NIBSC-19/108) in different drug dose ranges: (**a**) 0.01–100 µg/mL; (**b**) 0.063–1.6 µg/mL; (**c**) 0.001–0.5 µg/mL; and (**d**) 0.004–0.25 µg/mL.

In order to optimize the working drug dose concentration of the trastuzumab reference standard (NIBSC-19/108) in the SKBR3 cells, a series of broad and narrow concentration ranges were tested in a two-fold dilution series (Table 2). Following drug incubation, the fluorescence signals from the reduction in alamarBlue dye were recorded and the drug dose–response curve was plotted using the four-parameter logistic (4-PL) model (Figure 2). From the results, notably, the non-linear fitting curves for trastuzumab in the concentration dosage of 0.004–0.25 µg/mL exhibited improved signal-to-noise ratios (Figure 2d). Moreover, the selected concentration dose range showed better non-linear sigmoid curve fitting in terms of parallelism, linearity, and regression considering the parallel line analysis (PLA).

#### 3.2.2. Optimization of the Cell Seeding Density

**Figure 3:**
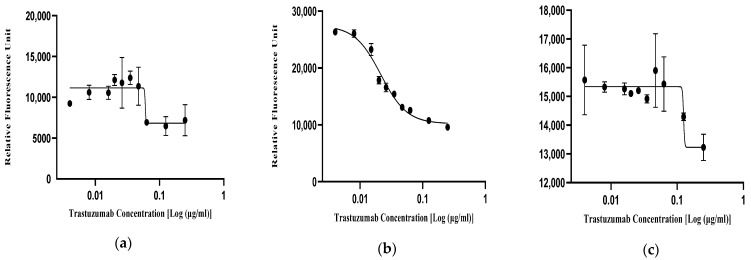
Optimization of the seeding density. (**a**) 5 × 10^3^, (**b**) 1.0 × 10^4^, and (**c**) 2.0 × 10^4^ cells per well were seeded to optimize the anti-proliferation assay for trastuzumab biosimilars. The 4-PL curves for the anti-HER2 reference standard (NIBSC-19/108) of different seeding densities.

In order to achieve optimal non-linear curve fitting for the selected drug dose range, three seeding densities were selected for the optimization in a 96-well plate format, i.e., 5 × 10^3^ cells/well, 1 × 10^4^ cells/well, and 2 × 10^4^ cells/well (Table 2). The optimized trastuzumab reference standard (NIBSC-19/108) dose concentration was added in duplicate and repeated thrice. A resazurin assay was performed to check the cellular viability of the cells after trastuzumab treatment. The relative fluorescence unit (RFU) of the resazurin dye directly correlated with the cell seeding density in the 96-well plate. Hence, based on the relative fluorescence units, the 4-PL curves of different cell densities were plotted (Figure 3) based on the optimized working drug dose range. The non-linear sigmoid curve, which showed good curve fitting at R^2^ > 0.98 with less % CV of <10%, was selected for the assay. Therefore, based on the 4-PL curve fitting, 1 × 10^4^ cells/well were selected, which provided an optimum fluorescence signal (Figure 3b).

#### 3.2.3. Optimization of the Incubation Time

**Figure 4:**
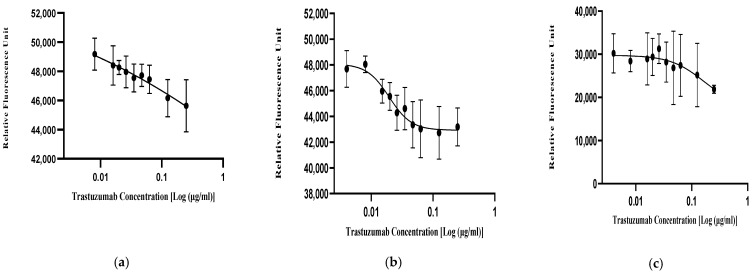
Optimization of the drug incubation time. 4-PL curves for the anti-HER2 reference standard (NIBSC 19/108) and trastuzumab biosimilar drug at different incubation times: (**a**) 96 h (4 days); (**b**) 120 h (5 days); and (**c**) 168 h (7 days).

The incubation time is crucial for a drug to function effectively. For this experiment, three distinct incubation times (96 h, 120 h, 168 h) (Table 2) were tested based on the doubling time of the SKBR3 cells with the trastuzumab reference standard (NIBSC-19/108) and were repeated thrice (Figure 4). From the results, it was observed that at 96 h (Figure 4a) and 168 h (Figure 4c), the drug did not show ideal curve fitting, whereas at 120 h, the optimized drug dose gave the ideal response with good curve fitting at R^2^ (>0.98) and less % CV (<10%). Hence, for the assay, 120 h were chosen as the optimal incubation time due to its higher maximal anti-proliferative activity based on the 4-PL non-linear sigmoid curve fitting (Figure 4b).

### 3.3. Validation of Inhibition of Proliferation (IOP) Assay

After the optimization, the cell-based bioassay needed to be validated based on the bioassay validation parameters according to the guidelines laid down by the US Pharmacopeia Chapters 1032, 1033, and 1034 as well as the ICH-Q2. The key parameters included specificity, linearity, precision, and accuracy.

#### 3.3.1. Specificity

**Figure 5:**
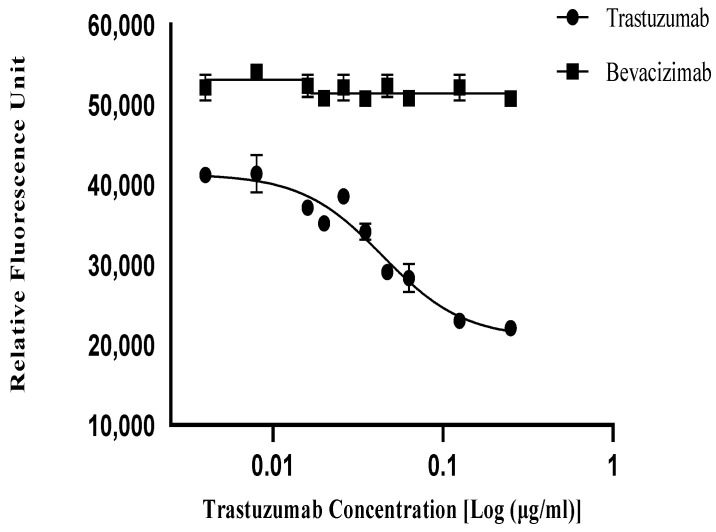
Specificity of the trastuzumab reference standard (NIBSC-19/108). The specificity of trastuzumab (NIBSC-19/108), where bevacizumab was used as a non-specific antibody, which did not show any dose response in the SKBR3 cell line.

To determine the specificity, the anti-proliferative activities of non-specific monoclonal antibodies of bevacizumab and the trastuzumab reference standard (NIBSC-19/108) targeting the HER2-positive cell line of SKBR3 were detected. The anti-proliferative activities of all the monoclonal antibodies were evaluated and subsequently compared. In the inhibition of proliferation assay, only trastuzumab could inhibit the activity of the HER2 receptor in a dose-dependent manner, and the non-specific mAbs did not affects the HER2 activity, indicating the specificity as well as stability of the anti-HER2 mAb of the established IOP bioassay (Figure 5).

#### 3.3.2. Linearity and Range

**Figure 6:**
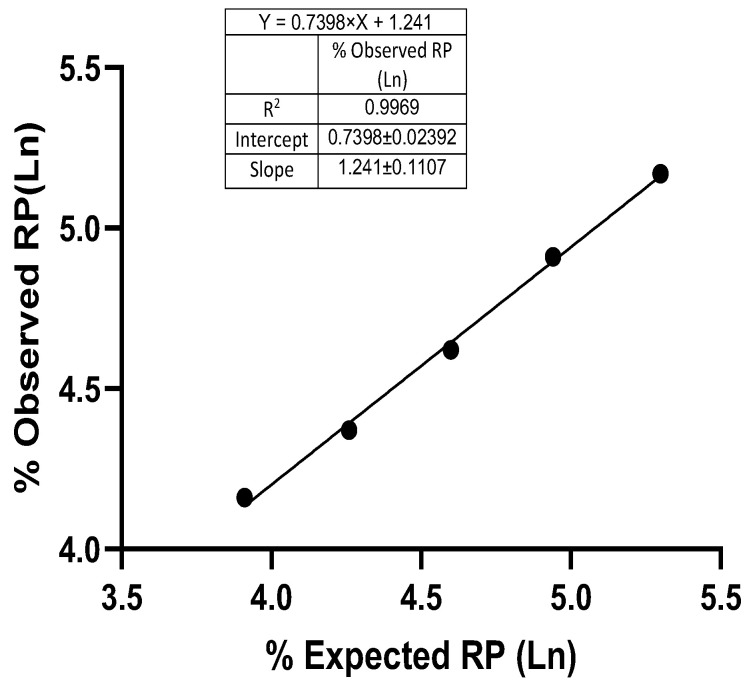
Linearity of the trastuzumab reference standard (NIBSC-19/108). Linearity of the inhibition of proliferation assay including the logarithmic relation between the observed and expected relative potencies.

For a potency assay, linearity ensures that the test results are directly proportional to the concentration of the drug in a sample within a specific range. For the assay, five independent samples of different concentrations were prepared by diluting the reference standard trastuzumab to achieve the simulated potencies of 50%, 71%, 100%, 141%, and 200%. A graph was plotted using the natural log (ln) of the observed % relative potencies, and the correlation coefficient (R^2^) value was found to be 0.99. The data were analyzed using a linear regression model to show the relationship between the observed and expected relative potencies (Figure 6). The range for the observed relative potency at each simulated level was found to be within the range of acceptable criteria of 80% to 125%.

#### 3.3.3. Precision and Repeatability

Precision refers to the level of agreement between measured values obtained from replicate measurements on identical or similar objects under specific conditions. Repeatability (intra-day precision) is an expression of the closeness of values taken under similar experimental conditions in a short span of time whereas intermediate precision (inter-day precision) is determined over a longer duration of time. The results are expressed as the Geometric Coefficient of Variation (% GCV) and should have been ≤20% for test samples (Table 3). For the inhibition of the proliferation assay, the repeatability (intra-day precision) was tested in triplicate and was found to be 1.21% (Table 4) and the intermediate precision was 19.58% (Table 3), which were within the range of the acceptable criteria for the bioassay.

#### 3.3.4. Accuracy

Accuracy is the expression of trueness and is defined as the measure of closeness of agreement between values that are accepted either as an accepted reference value or the value estimated. The average percentage recovery should fall within the range of 80% to 125%. This is expressed as a percentage relative bias which should be less than 5%, highlighting the importance of closeness of agreement between the accepted and estimated values. For the inhibition of proliferation assay, the % relative bias was found to be 0.0067% (Table 3).

### 3.4. Comparison of the Biological Activity of the Trastuzumab Biosimilar Drugs

Under the optimized and validated experimental conditions, the bioactivities of four trastuzumab biosimilars were compared with the reference standard drug for trastuzumab (NIBSC-19/108) in the SKBR3 cell line. The relative potencies of the biosimilar drugs were compared for the inhibition of proliferation assay and the flow cytometry binding assay to assess the similarity of the trastuzumab biosimilar drugs to the trastuzumab reference standard (NIBSC-19/108). In order to analyze the relative potencies, the four-parameter logistic (4-PL) curves of the bioactivities of the trastuzumab biosimilars were evaluated.

#### 3.4.1. Based on the Inhibition of Proliferation (IOP) Assay

**Figure 7:**
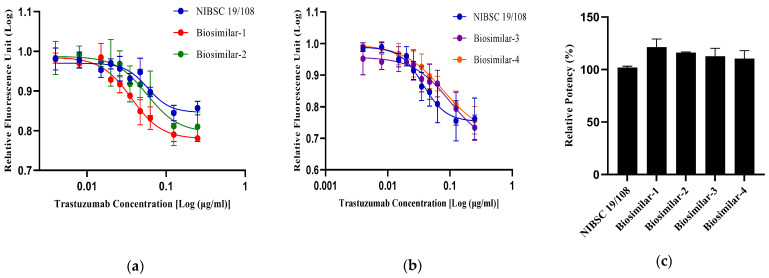
Comparing the anti-proliferative activity of trastuzumab biosimilars with the alamarBlue cell viability assay. (**a**,**b**) 4-PL curves for the anti-HER2 reference standard (NIBSC-19/108) and trastuzumab biosimilar drug were measured using the alamarBlue proliferation inhibition assay in the HER2+ breast cancer cell line of SKBR-3. Experiments were performed in triplicate and for 120 h of treatment. (**c**) % Relative potencies of four biosimilar drugs with respect to the trastuzumab reference standard (NIBSC-19/108). Data are represented as the mean ± SD.

The anti-proliferative activity of the trastuzumab reference standard (NIBSC-19/108) and various biosimilars of trastuzumab were evaluated using the resazurin (alamarBlue) proliferation inhibition assay in the HER2-positive breast cancer cell line of SKBR3. The experiment was conducted in a 96-well plate and performed in triplicate. Based on the fluorescent signals, 4-PL curve fitting was performed (Figure 7a,b) for all biosimilars and the reference standard drug. It was found that the curves fitted with similar R^2^ and low % CV values. Furthermore, the relative potencies of the reference standard drug and biosimilars were evaluated and compared via parallel line analysis (PLA). The results showed that the relative potencies of all the trastuzumab biosimilar and reference standard drug were within the acceptable range of 80–125% based on various national and international guidelines, where the relative potencies for NIBSC-19/108 was 101.9%, Biosimilar-1 was 121.3%, Biosimilar-2 was 116.1%, Biosimilar-3 was 112.7%, and Biosimilar-4 was 110.5% (Figure 7c). There was no statistical difference between the relative potencies of the trastuzumab reference standard (NIBSC-19/108) and biosimilar drugs evaluated by ANOVA (Dunnett’s multiple comparisons test). Hence, it can be inferred that there was no significant difference between the bioactivity of the reference standard drug and its biosimilars.

#### 3.4.2. Based on the Binding Assay by Flow Cytometry

**Figure 8:**
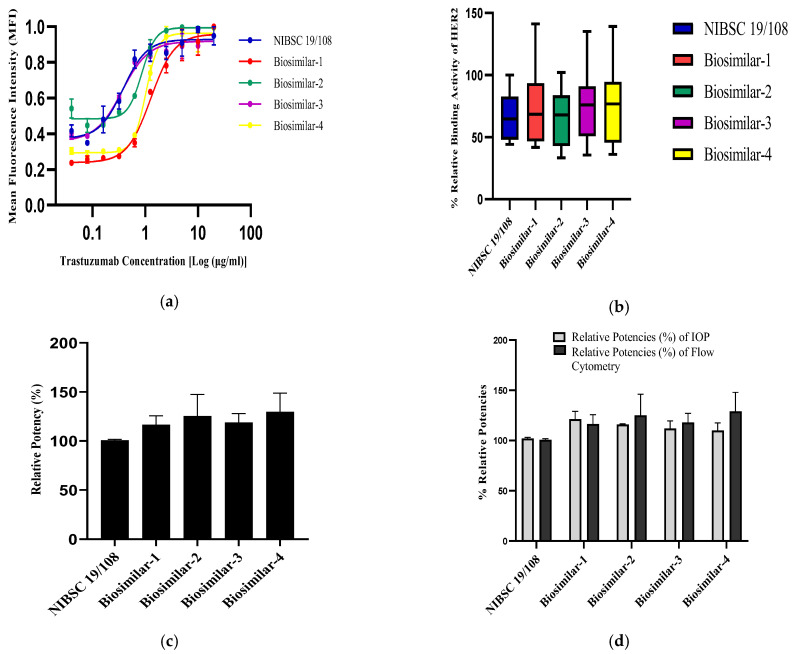
HER2 binding assay by flow cytometry: (**a**) 4-PL curve for anti-HER2 trastuzumab reference (NIBSC-19/108) and trastuzumab biosimilar drug measured by flow cytometry; (**b**) % Relative binding activity of trastuzumab/reference product and four trastuzumab biosimilars with the HER2 receptor; (**c**) % Relative potencies of four biosimilar drugs with respect to the trastuzumab reference standard (NIBSC-19/108). Data are represented as the mean ± SD. (**d**) % Relative potencies evaluated from the IOP and flow cytometry. Data are represented as the mean ± 2SD.

The binding of the trastuzumab reference standard and its biosimilars to the HER2 receptor was conducted in the SKBR3 cells, where the cells were incubated with the reference standard and biosimilars of trastuzumab in two-fold serial dilutions ranging from 20 µg/mL to 0.04 µg/mL. The mean fluorescence intensity (MFI) was recorded for both the reference standard (NIBSC-19/108) and different biosimilars of trastuzumab and the 4-PL sigmoid curve was plotted (Figure 8a), where all the biosimilars as well as the reference standard of trastuzumab exhibited good R^2^ fitting with less % CV. Additionally, the HER2 binding of all the trastuzumab drugs was also assessed, where it demonstrated that the reference standard and the biosimilars of trastuzumab bound to the HER2 receptor with similar affinity and no significant difference, as evaluated by ANOVA (Dunnett’s multiple comparisons test) (Figure 8b). Furthermore, parallel line analysis (PLA) was conducted to determine the relative potencies of the biosimilars drugs with respect to the reference standard of trastuzumab. It was found that the relative potencies of all the various biosimilars of trastuzumab were within the acceptable range of 80–125% as per the national and international guidelines. The relative potency of the reference standard was 100.7%, Biosimilar-1 was 123%, Biosimilar-2 was 109.9%, Biosimilar-3 was 123.5% and Biosimilar-4 was 116.2% (Figure 8c). There was no statistical difference between the relative potencies of the trastuzumab reference standard (NIBSC-19/108) and biosimilar drugs evaluated by ANOVA (Dunnett’s multiple comparisons test). Hence, it can be inferred that there was no significant difference between the bioactivity as well as the binding of the reference standard drug and its biosimilars.

Table 5 and Figure 8d illustrate that the quantitative results of the % relative potencies, as determined from both the assays of inhibition of proliferation (IOP) and flow cytometry, were identical in terms of potency and binding, with no statistically significant difference found between the two tests, as determined by a two-tailed paired *t*-test (Wilcoxon matched-pairs signed-rank test). Therefore, it can be inferred that inhibiting proliferation is a reliable assay for assessing the bioactivity of trastuzumab biosimilar drugs using SKBR3 cells. These drugs attach to the HER2 receptor present on the cell membrane of SKBR3 cells with a similar affinity and mechanism as the reference standard (NIBSC-19/108) of trastuzumab, with consistent potency measurements observed for trastuzumab biosimilars in the SKBR3 cells.

## 4. Discussion

One of the critical quality attributes (CQAs) of a biosimilar monoclonal antibody drug that can demonstrate its therapeutic equivalence to the reference originator product is its biological activity [13]. The biological activity of a biosimilar should exhibit a similar binding affinity to the target antigen and comparable pharmacological effects, including in its potency and mechanism of action (MOA) [21]. Various studies involving the development of bioassays for mAbs have been conducted previously, where the assessment of biological activity of vedolizumab was performed by the cell-based ELISA method, which was found to be reliable, yielding results similar to flow cytometry (70–130% relative potency). It showed high linearity (R^2^ = 0.99) between 50 and 200%, proving it to be a cost-effective alternative for quality control [22]. Similarly, another study developed a reliable fluorescence-based complement-dependent cytotoxicity assay for anti-T lymphocyte immunoglobulin, demonstrating high specificity, linearity, repeatability, and accuracy within established compendial limits, showcasing compliance with USP and ICH guidelines [13]. However, while developing a cell-based potency assay to evaluate the biological activity of a biologic, selecting a suitable cell line is important as it should respond specifically to the biologic being tested without notable interference from other factors present in the assay system as well as impart similar therapeutic equivalence as that of the originator biological molecule [23]. For the optimization and validation of cell-based potency assay of trastuzumab biosimilars, the selection of a cell line was based on the surface-level expression of HER2, molecular expression of the HER2/ERBB2 gene, and binding affinity of the biosimilar molecule to the HER2 target on the cell line. The monoclonal antibody trastuzumab binds to the HER2 receptor and is expressed abundantly on the breast cancer cell line surface [24], and it causes a reduction in the dose-dependent growth of HER2-positive breast cancer cells [25]. The HER2 receptor expression in SKBR3 was found to be almost ten times higher than BT474, wherein SKBR3 cells were reported to quantitatively express 1.5 × 10^6^ HER2 receptors on their surface, whereas BT474 cells expressed 1.5 × 10^5^ HER2 receptors on their cell surface [26,27]. This study found that the cell surface-level expression of the HER2 receptor is high in SKBR3 and BT474 followed by MDA-MB-453 cells from the immunoblotting analysis. However, the gene expression and flow cytometry analysis of the HER2 receptor on various cell lines in this study further revealed that SKBR3 cells expressed the HER2 receptor relatively higher than the BT474 and MDA-MB-453 cells. Previously, flow cytometry analysis for the cell surface level expression of the HER2 receptor revealed that SKBR3 expressed the HER2 receptor relatively more than BT474 [28]. The RNA expression values of the ERBB2 gene in 30 breast cancer cell line panels demonstrated that SKBR3 expresses 3046 TPM (transcripts per million), BT474 expresses 2153 TPM, and MDA-MB-453 expresses 446 TPM, making SKBR3 more pronounced in expressing the ERBB2/HER2 gene than the other cell lines [29]. The SKBR3 cells demonstrated higher sensitivity to trastuzumab treatment compared to BT474 cells in an in vitro study, wherein SKBR3 cells were demonstrated to be more reactive to trastuzumab treatment than BT474 cells [30]. The expression of HER2 receptors obtained in SKBR3 cells in the present study was in line with the previous literature, and therefore, SRBR3 cells were chosen for further developing a cell-based potency assay for trastuzumab biosimilars.

The International Council for Harmonisation (ICH) defines potency as the quantitative measure of a product’s biological activity based on its relevant biological properties [31]. According to ICH Q6B guidelines, most of the potency assays for biologics are either cell-based and/or biochemical assays which correlate to the biological activity of the biosimilar drugs [31]. An ideal potency assay should be able to replicate a biological drug’s mechanism of action (MOA) in an in vitro setting and perform consistently in quality control environments. Moreover, the potency assay needs to be extensively validated and optimized as per international guidelines to ensure its reliability and accuracy in terms of critical assay parameters of specificity, linearity, detection range, precision, and accuracy [13,14]. Hence, to effectively evaluate the similarity between test and reference biological drugs, it is essential to optimize and validate the potency of cell-based bioassays. To demonstrate biosimilarity, measuring the relative potency of a test and reference biological drug has become a critical component in quality control settings, thus ensuring the safe and effective use of biosimilars in clinical practice. The penultimate objective of our study was to optimize the trastuzumab biosimilar dose–response curve for the inhibition of a proliferation assay using the SKBR3 cell line. For this study, a cell-based inhibition of proliferation assay was selected to assess the biological activity of the biologic. This bioassay is notably economical, robust, and highly reproducible. For optimization of the bioassay, one parameter was changed while the others were kept constant. The cell seeding density was optimized at 1 × 10^4^ SKBR3 cells per 96 wells based on the 4-PL curve fitting analysis with R^2^ > 0.98 and % CV < 10%, and the incubation time for the assay was 120 h, considering the doubling time of the SKBR3 cell line and the 4-PL curve fitting analysis. The optimal dose concentration for both the biosimilar and trastuzumab reference standard was found to be 0.004–0.25 µg/mL, based on the best sigmoid curve fitting with R^2^ > 0.98. Furthermore, for the validation of the anti-proliferative bioassay for the trastuzumab biosimilar drug, the bioassay performance characteristics, including specificity, precision, accuracy, linearity, and range were taken into consideration for this study. The IOP assay using the SKBR3 cell line under optimized conditions was thus formed and validated for the estimation of the biological activity of trastuzumab and its biosimilars with high specificity, precision (% RSD), accuracy (% GCV = 19.1%), linearity (R^2^ ≥ 1.0) and range (50–200%).

Finally, the biosimilarity of four trastuzumab biosimilars and reference standard (NIBSC-19/108) based on the optimized and validated IOP bioassay was established and the HER2 binding activity of the trastuzumab biosimilars showed similarity to the reference standard product (NIBSC-19/108). The relative potencies for the trastuzumab biosimilars were similar to the reference standard drug and were within the range of acceptable criteria of 80–125%. Moreover, the HER2 binding activities of all trastuzumab biosimilars were the same as the reference standard drug, with no statistically significant difference, exhibiting a favorable resemblance in HER2 binding activity. For the inhibition of proliferation bioassay, the % relative potency quantified in terms of the relative fluorescence unit (RFU) for trastuzumab biosimilars and reference standard drugs was within the acceptance criteria defined by international agencies, of ranges from 80 to 125% with confidence intervals (CIs) of 95%. The biosimilars exhibited relative potencies within the permissible range and thus demonstrated promising similarity in the inhibition of the proliferation potency bioassay. Hence, the present study indicated that the trastuzumab biosimilars were equivalent to the trastuzumab reference standard (NIBSC-19/108) concerning the biological characteristics associated with antigen-binding fragments (Fab). Consequently, the inhibition of proliferation assay can be employed in routine quality control testing to evaluate the bioactivity, including the binding and potency, of trastuzumab, its biosimilars, and its biobetters. The caveat of this study was that the bioassay method exclusively evaluated the cellular phenotypic response for the inhibition of cell proliferation to assess the bioactivity of trastuzumab biosimilars based on its binding to HER2 receptors, which was relatively more cost-effective, reliable, and robust than other orthogonal bioassays (ELISA, flow cytometry, reporter gene assays) for the functional characterization of trastuzumab-like antibodies. Furthermore, IOP bioassay needs to be supported by integrating various functional assays, such as antibody-dependent cellular cytotoxicity (ADCC), antibody-dependent phagocytosis (ADPC), and apoptosis assays [32], to ensure the overall therapeutic efficacy of trastuzumab and its biosimilars during developmental and regulatory testing before market authorization.

## 5. Conclusions

In conclusion, the present investigation suggested the harmonization of the inhibition of proliferation assay for the estimation of the biological activity of the trastuzumab and its biosimilars by employing SKBR3 cells. The present study was unique wherein the expression of the HER2 receptor was considered a critical quality attribute for the optimization and validation of the bioassay for trastuzumab and its biosimilars, as well as the binding of trastuzumab drugs to the HER2 receptor. The assay was duly validated for the method validation parameters as per various international regulatory guidelines, including specificity, precision, accuracy, and linearity. The developed inhibition of proliferation assay will help ensure the quality of trastuzumab and its biosimilars during product development, comparability exercises, batch release testing, stability, and post-market surveillance.

## Figures and Tables

**Table 1:** List of trastuzumab-like antibodies used in this study.

Product/Brand Name	Manufacturer
1st WHO International Standard (NIBSC-19/108) for the biological activity of trastuzumab	NIBSC, UK
CANMab	M/s Biocon India Pvt. Ltd., Bangalore, Karnataka, India
TrastuRel	M/s Reliance Life Sciences, Mumbai, MH, India
Eleftha	M/s Intas Pharmaceuticals Ltd., Ahmedabad, GJ, India
Vivitra	M/s Zydus Ahmedabad, GJ, India

**Table 2:** Assay optimization parameters.

Parameter	Experimental Conditions	Optimized Conditions
Working drug dose range	0.01–100 µg/mL, 0.063–1.6 µg/mL, 0.001–0.5 µg/mL, and 0.004–0.25 µg/mL	0.004–0.25 µg/mL
Seeding density	5.0 × 10^3^, 1.0 × 10^4^ and 2.0 × 10^4^ cells per well	1.0 × 10^4^ cells per well in 96-well plate
Drug incubation time	96 h, 120 h, 168 h	120 h

**Table 3:** Evaluation of intermediate precision and accuracy calculations for the inhibition of proliferation assay.

Analyst	Analyst 1			Analyst 2			
Performance	Performance 1	Performance 2	Performance 3	Performance 1	Performance 2	Performance 3	Performance 4
Relative Potency (RP)	122	120.7	119.1	92.6	91.7	81	83.4
Ln (RP)	4.80	4.79	4.77	4.52	4.51	4.39	0.42
Mean				4.62			
SD				0.17			
GM				100.01			
% GCV				19.58			
% Relative Bias				0.0067			

**Table 4:** Evaluation of the repeatability of the inhibition of proliferation assay.

Analyst	Performance	Relative Potency (RP)	Ln (RP)	Mean (Ln RP)	SD (Ln RP)	% GCV
Analyst 1	Performance 1	122	4.80	4.79	0.012	1.21
Analyst 1	Performance 2	120.7	4.79			
Analyst 1	Performance 3	119.1	4.77			

**Table 5:** Quantitative analysis of the % relative potencies of trastuzumab biosimilars in SKBR-3.

	Relative Potency (%) of IOP	Relative Potency (%) of Flow Cytometry
NIBSC-19/108	101.9667	100.7
Biosimilar 1	121.3	116.45
Biosimilar 2	116.1	125.45
Biosimilar 3	112.7	118.9
Biosimilar 4	110.4633	129.65

## Data Availability

The original contributions presented in this study are included in this article; further inquiries can be directed to the corresponding author.
